# SIRT3 alleviates imiquimod-induced psoriatic dermatitis through deacetylation of XBP1s and modulation of TLR7/8 inducing IL-23 production in macrophages

**DOI:** 10.3389/fimmu.2023.1128543

**Published:** 2023-05-19

**Authors:** Meiliang Guo, Haojun Zhuang, Yimin Su, Qinqin Meng, Wanwen Liu, Na Liu, Min Wei, Sheng-Ming Dai, Hui Deng

**Affiliations:** ^1^ Department of Dermatology, Shanghai Sixth People’s Hospital Affiliated to Shanghai Jiao Tong University School of Medicine, Shanghai, China; ^2^ Department of Rheumatology & Immunology, Shanghai Sixth People’s Hospital Affiliated to Shanghai Jiao Tong University School of Medicine, Shanghai, China

**Keywords:** psoriasis, macrophage, IL-23, SIRT3, XBP1s

## Abstract

Current evidence suggests that IL-23, IL-6, and TNF-α play pivotal roles in the pathogenesis of psoriasis. Although it has been established that Sirtuin 3 (SIRT3) mediates the inflammatory process, the underlying mechanisms remain largely unclear. Herein, we substantiated that the inhibition or deletion of SIRT3 increased the acetylation level of spliced form of X-box binding protein 1 (XPB1s), enhancing its transcriptional activity and IL-23a production. Pharmacologically inhibition of XBP1s with MKC8866 downregulated the expression of inflammatory cytokines in SIRT3-inhibited or *Sirt3*-KO BMDMs stimulated by IMQ. Inhibition or knockdown of SIRT3 could exacerbate psoriasis-like skin inflammation in an imiquimod-induced psoriasis-like mouse model. Besides, a decrease in SIRT3 expression was observed in the macrophages of psoriasis patients, which increased the expression and acetylation level of XBP1s. Overall, we provide compelling evidence of the crucial role of SIRT3 in the IL-23 axis in psoriatic inflammation and novel molecular insights into the anti-inflammatory effects of SIRT3.

## 1 Introduction

Psoriasis is an immune-mediated, chronic disease that affects 125 million people worldwide, leading to a decrease in their quality of life and is associated with several comorbidities (1). Interleukin-23 (IL-23) and tumor necrosis factor α (TNF-α) play pivotal roles in psoriasis, and their corresponding inhibitors are recommended for first-line treatment of moderate to severe plaque psoriasis by the American Academy of Dermatology-National Psoriasis Foundation guidelines (2).

The unfolded protein response (UPR) is an evolutionarily conserved response triggered by the accumulation of unfolded/misfolded proteins that aims to maintain cellular homeostasis (3). Recent evidence suggests that UPR plays a crucial role in immunity and inflammation progress (4). The activation of the unfolded protein response (UPR) in macrophages is triggered by the Toll-like receptors (TLRs) signal, which amplifies the TLR response *via* IRE1α-induced XBP1 splicing and the deletion of spliced form of X-box binding protein 1 (XPB1s) results in decreasing production of innate immune mediators (4–6). As a key downstream signaling molecule for UPR, XBP1s interacts directly with the *Il23*a**, *Il6 * and *Tnf* gene promoters. Subsequently, TLR-mediated UPR activation increases cytokine expression through the XBP1 pathways and thus exacerbates TLR-mediated inflammation (4, 5). Growing evidence suggests that deacetylation of XBP1s by Sirtuins impairs its transcriptional activity and protein stability, whereas acetylation of XBP1s promotes UPR and exacerbates the exacerbation of inflammation (7). Although Sirtuin 3 (SIRT3) has been recognized as a protein that mainly governs lysine acetylation in mitochondria, there is an increasing consensus that SIRT3 is also present in the nucleus. In a previous study, SIRT3 was found to be downregulated in psoriatic lesions (8). Although SIRT3 is reportedly crucial for the mitochondrial unfolded protein response (mtUPR), which is reportedly activated by stressful events as a stress-protective response, its involvement in UPR remains unexplored (9).

Here, we focused on the deacetylation activity of SIRT3 in the UPR in psoriasis. We hypothesized that SIRT3 diminished TLR7/8-XBP1s response and thereby ameliorated inflammation in psoriasis. Our findings indicate that suppressing SIRT3 exacerbated IMQ-induced psoriasis-like inflammation by upregulating the levels of acetylated XBP1s, which subsequently enhanced its total protein levels and transcriptional activity and increased the production of IL-23a, IL-6, and TNF-α cytokines.

## 2 Materials and methods

### 2.1 Human samples

We enrolled 50 individuals with a confirmed diagnosis of psoriasis based on pathological examination and 50 age- and sex-matched healthy controls from outpatient clinics in Shanghai Sixth People’s Hospital Affiliated to Shanghai Jiao Tong University School of Medicine in the study. The Medical Ethics Committee of Shanghai Sixth People’s Hospital reviewed and approved the protocol, and all patients provided written informed consent.

### 2.2 Cell isolation and culture

Monocyte-derived macrophages and dendritic cells(DC) were generated from peripheral blood mononuclear cells (PBMCs) as described previously (10, 11). Briefly, PBMCs were isolated from the peripheral blood by density gradient centrifugation. For monocyte-derived macrophages, PBMCs were differentiated by culturing for 7 days in RPMI 1640 medium (Gibco, CA) with 10% fetal bovine serum(FBS) (Gibco, CA), 1% antibiotics(Yeasen, Shanghai, China) and 50 ng/ml Human macrophages colony-stimulating factor (M-CSF, PeproTech, USA) at 37°C and 5% CO_2_. For monocyte-derived DC, PBMCs were cultured in medium supplemented with 100 ng/ml IL-4 (PeproTech, USA) and 100 ng/ml granulocyte macrophage colony-stimulating factor(GM-CSF, PeproTech, USA). Bone marrow-derived macrophages (BMDMs) differentiated in the presence of 20 ng/ml Mouse M-CSF (PeproTech, USA) were isolated from femurs of WT and *Sirt3^-/-^* C57/BL6 mice and cultured in RPMI 1640 medium containing 10% FBS and used at day 8. The human embryonic kidney cell line HEK293T was obtained from the Diabetes Institute of Shanghai Jiao Tong University Affiliated Sixth People’s Hospital (Shanghai, China) and cultured in Dulbecco’s modified Eagle’s medium containing 10% FBS and 1% of penicillin-streptomycin.

### 2.3 Antibodies and reagents

Antibodies against XBP1s and Ac-K(Acetylated-Lysine) were obtained from Cell Signaling Technology (Danvers, MA, USA), and anti-SIRT3 antibodies were obtained from Proteintech (Wuhan, China) and Everest Biotech (Oxfordshire, UK). Donkey Anti-Rabbt IgG H&L (Alexa Fluor 488) and Donkey Anti-Mouse IgG H&L (Alexa Fluor 647) were obtained from Abcam (Cambridge, MA, USA). Anti-glyceraldehyde 3-phosphate dehydrogenase (GAPDH) was purchased from Boster Bio (Wuhan, China). FITC anti-human CD3 Antibody, APC anti-human CD14 Antibody were obtained from BioLegend(San Diego, CA). The IMQ powder, MKC8866, 3-TYP and Honokiol were from MedChemExpress (Princeton, NJ, USA). IMQ cream was obtained from Sichuan Med-Shine Pharmaceutical Co., Ltd. (Sichuan, China).

### 2.4 Immunoblotting and co-immunoprecipitation

Lysis buffer was applied to obtain the protein samples, separated by 10% SDS-PAGE, and transferred to PVDF membranes. The membranes were blocked with 5% skim milk, incubated with primary antibodies to XBP1s, SIRT3, Ac-K, and GAPDH overnight at 4°C, and then incubated with secondary antibodies. The intensity of the bands was measured using ImageQuant LAS chemiluminescence imager (GE Healthcare Life Sciences). For the co-immunoprecipitation assay, minimal amounts of antibody or normal IgG were added to 1 mg cell lysates precleared with Protein A/G-Plus Beads (Yerk, Shanghai, China) and then incubated at 4°C overnight. Protein A/G-Plus Beads were added and incubated for 3 hours at 4°C. Blots were probed with primary antibodies against Acetylated-lysine, SIRT3, or XBP1s, and antibody signals were detected.

### 2.5 Plasmids and transfection

Flag-XBP1s, XBP1s, Flag-SIRT3, and SIRT3 constructs were obtained from iGenbio (Guangzhou, China). p300 construct was kindly provided by the Li Jingya research group (Shanghai Institute of Pharmacy, Chinese Academy of Sciences). Cell transfection was performed using Lipofectamine 2000 (Invitrogen, Carlsbad, CA, USA).

### 2.6 Immunofluorescence

Cells were fixed in 4% paraformaldehyde and permeabilized with 0.2% Triton X-100. After blocking with 5% BSA solution for 1 h at room temperature (RT), cells were incubated with primary antibodies (XBP1s, SIRT3) diluted in 1% BSA solution. The next day, cells were incubated with AlexaFluor 488-and 647-conjugated secondary antibodies and DAPI for 1h at RT. Images were visualized by a confocal fluorescent microscope (Leica TCS SP8, Germany).

### 2.7 Real-Time Quantitative Reverse Transcription Polymerase Chain Reaction (qRT-PCR)

Total RNA was extracted from cells or fresh skin tissue using EZB-press RNA Purification Kit (EZBioscience, Shanghai, China). The ABScript II RT Mix for qPCR with gDNA Remover (ABcnonal, Wuhan, China) was used to synthesize cDNA. The qPCR reactions mixture comprised cDNA, forward and reverse primers, and PCR master mixture (ABcnonal, Wuhan, China). The qPCR reaction was performed in 96-well plates on a QuantStudio7 Real-time detection system. Primers used for real-time PCR are listed in **Table 1**.

**Table 1:** Sequences for primers used in Real-time RT-PCR.

Prime name	Sequence(5’ to 3’ )
*I123a* forward primer	ATGCTGGATTGCAGAGCAGTA
*Il23a* reverse primer	ACGGGGCACATTATTTTTAGTCT
*Il6* forward primer	TACCACTTCACAAGTCGGAGGC
*Il6* reverse primer	CTGCAAGTGCATCATCGTTGTTC
*Tnfa* forward primer	GGTGCCTATGTCTCAGCCTCTT
*Tnfa* reverse primer	GCCATAGAACTGATGAGAGGGAG
*Gapdh* forward primer	GGAGCGAGATCCCTCCAAAAT
*Gapdh* reverse primer	GGCTGTTGTCATACTTCTCATGG
*SIRT3* forward primer	CCCTGGAAACTACAAGCCCAAC
*SIRT3* reverse primer	GCAGAGGCAAAGGTTCCATGAG
*GAPDH* forward primer	GGAGCGAGATCCCTCCAAAAT
*GAPDH* reverse primer	GGCTGTTGTCATACTTCTCATGG

### 2.8 Animals and treatments

C57BL/6 mice were obtained and maintained under specific pathogen-free conditions in the Shanghai Jiaotong University Affiliated Sixth People’s Hospital Animal Department. *Sirt3* ^-/-^ mice on a C57BL/6 background were obtained from Cyagen (Cyagen Biosciences Inc., China). For the 3-TYP group, C57BL/6 mice were pretreated with 3-TYP (at a dose of 50 mg/kg ip every 2 days) and continued for 7 d after IMQ cream application. For the Honokiol group, C57BL/6 mice were pretreated with Honokiol (10 mg/kg/d ip) and continued for 7 d after IMQ cream application. For the psoriasis-like mouse model, we applied a daily topical dose of 62.5 mg of IMQ cream (Sichuan Med-Shine Pharmaceutical Co., Ltd., Sichuan, China) on the shaved back for 7 consecutive days. The back lesions inflammation severity of the mouse model was measured daily using a modified human PASI scoring system. The animals were sacrificed on day 7 after IMQ treatment.

### 2.9 Histological analysis

The back skin of mice was fixed in formalin, embedded into paraffin, and sliced into thin sections. The sections were stained with hematoxylin and eosin and examined by microscopy.

### 2.10 Flow cytometry cell sorting

To prepare the cells for flow cytometry sorting, PBMCs were resuspended in RPMI 1640 medium with 10% fetal bovine serum and incubated with fluorochrome-conjugated CD3(FITC) and CD14(APC) antibodies for 60 min at room temperature. Cell sorting was performed with a MoFlo XDP Cell Sorter (Beckman Coulter).

### 2.11 Statistical analysis

Data are presented as mean ± SEM. An Independent two-sample Student’s t-test was performed to assess comparisons between 2 groups, and analysis of variance was applied for multiple comparisons. Statistical analysis was performed on GraphPad Prism 8 (GraphPad Software, San Diego, CA, USA). 

## 3 Results

### 3.1 Pharmacological inhibition of SIRT3 upregulates XBP1s expression and TLR7/8 inducing IL-23A production

**Figure 1:**
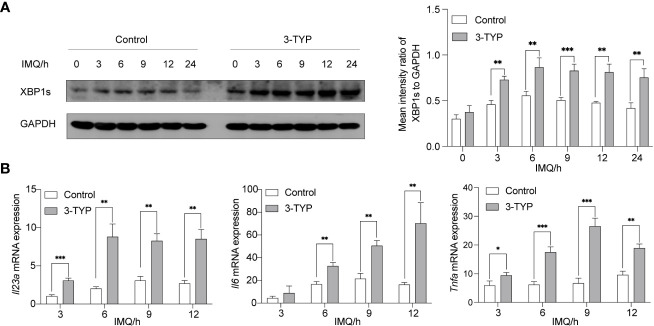
Inhibition of SIRT3 aggravated the expression of XBP1s and the production of *Il23a* mRNA. **(A)** C57BL/6 mice-derived BMDMs pretreated with or without 50μM 3-TYP for 1 hour were stimulated with 20μM IMQ for 0, 3, 6, and 9 hours and cell lysates were immunoblotted for XBP1s and GAPDH. **(B)** mRNA levels of inflammatory cytokines were determined by qRT-PCR. Values are expressed as mean ± SEM. *: P<0.05, **: P<0.01, ***: P<0.001. IMQ, imiquimod.

It is now understood that XBP1s is the functional protein of XBP1 mRNA activated by an IRE1-dependent unconventional cytosolic mRNA-splicing event. Studies have demonstrated that TLR4 and TLR2 specifically promoted cytosolic splicing of the transcription factor XBP1s (5). Consistently, we revealed that the activation of XBP1s in BMDMs was triggered upon stimulation of TLR7/8. Under the same conditions, we examined whether pharmacological inhibition of SIRT3 regulates the IMQ-mediated activation of XBP1s. 3-TYP is a selective SIRT3 inhibitor that does not affect SIRT3 protein expression. As shown in **Figure 1A**, 50µM 3-TYP significantly increased the production of XBP1s. Next, we explored the expression of SIRT3 upon TLR7/8 activation and found that SIRT3 expression was relatively stable within 12 hours, but would decrease in 18 hours(**Supplementary Figure**). To further examine the effects of SIRT3 on the TLR-XBP1s response, we analyzed the XBP1s-dependent transcription levels of *Il23*a*, Il6* and *Tnf*a**. Co-treatment with IMQ and 3-TYP resulted in significant upregulation of *Il23*a*, Il6* and *Tnf*a** mRNA levels (**Figure 1B**). Taken together, these data indicated that SIRT3 inhibition markedly potentiated the TLR-XBP1s response and the production of downstream inflammatory factors.

### 3.2 SIRT3 regulates XBP1s expression by mediating its deacetylation activity

**Figure 2:**
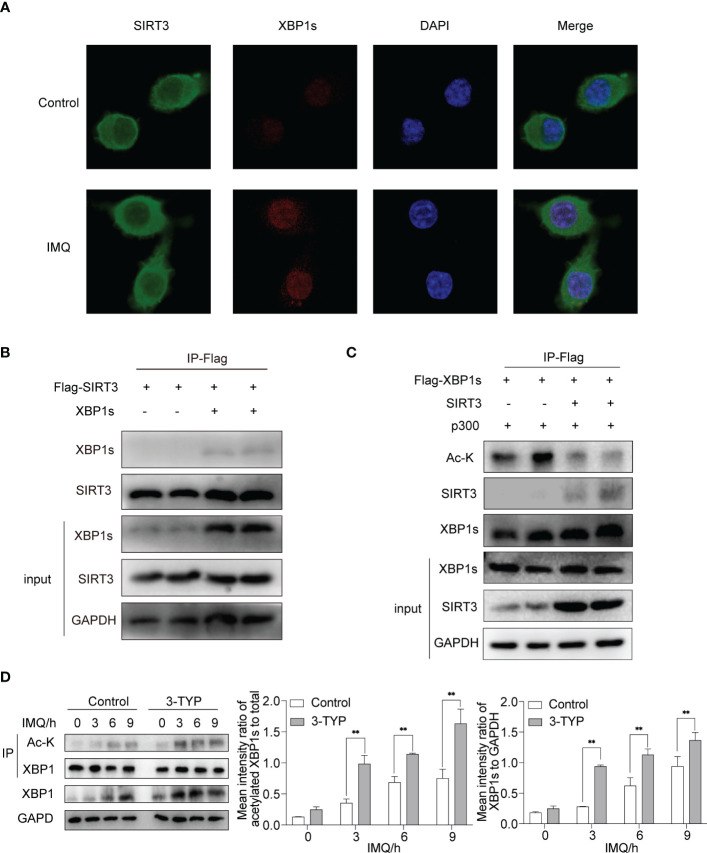
SIRT3 interacted with XBP1s and regulated XBP1s level through deacetylation activity. **(A)** Fluorescence analysis of SIRT3 and XBP1s in BMDMs stimulated by IMQ for 12 hours. **(B)** HEK293T cells were transfected with indicated plasmids, and the interactions between SIRT3 and XBP1s were analyzed. **(C)** The deacetylation effect of SIRT3 on XBP1s was analyzed in HEK293T. **(D)** BMDMs pretreated with or without 50μM 3-TYP for 1 hour were stimulated with 20μM IMQ for 0, 3, 6, and 9 hours and total cell lysates were immunoprecipitated with anti-XBP1s antibodies and immunoblotted with anti-Ac-k antibodies. Values are expressed as mean ± SEM. **: P<0.01. IMQ, imiquimod; Ac-K, Acetylated-Lysine; HEK293T, Human embryonic kidney cell line HEK293T; IP, immunoprecipitation.

Current evidence suggests that the acetylation status of XBP1s affects its stability and thus changes its level (7, 12, 13). Accordingly, we investigated the molecular mechanism of SIRT3 regulating XBP1s level, focusing on the deacetylase activity of SIRT3. We performed a co-immunofluorescence experiment to study the intracellular localization of SIRT3 and XBP1s in macrophages. To determine the localization of both proteins, BMDMs were double-labeled with primary antibodies against SIRT3 and XBP1s and then visualized under a confocal microscope after being stimulated with or without IMQ. Consistent with the literature (14), we found that SIRT3 was predominantly present in the cytoplasm, with low expression in the nucleus. XBP1s expression was barely detectable under resting conditions, and after stimulating with IMQ, it was expressed in the nucleus. SIRT3 and XBP1s were localized within the nucleus, while TLR7/8 was activated (**Figure 2A**).

We performed co-immunoprecipitation experiments to further investigate the association between SIRT3 and XBP1s. We co-expressed Flag-SIRT3 with or without XBP1s in HEK293T cells. The co-IP experiment demonstrated direct binding between SIRT3 and XBP1s (**Figure 2B**). Next, we assessed the effect of SIRT3 activity on the acetylation levels of XBP1s. When SIRT3 was overexpressed in 293T cells, XBP1s acetylation significantly decreased (**Figure 2C**). In addition, we verified the deacetylation ability of SIRT3 in BMDMs. Stimulation of BMDMs with IMQ following treatment with 3-TYP resulted in elevated expression and acetylation of XBP1s compared to the control group (**Figure 2D**), which indicated that SIRT3 functions as a deacetylase for XBP1s. These findings suggest that XBP1s serve as a substrate for SIRT3, and SIRT3 curtails the transcriptional activity of XBP1s toward *Il23*a*, Il6* and *Tnf*a** through its deacetylation function.

### 3.3 Deletion of *Sirt3* leads to exacerbated increased XBP1s acetylation status and TLR7/8 response

**Figure 3:**
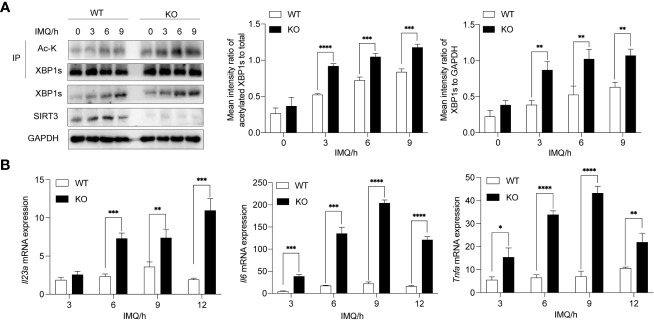
Deletion of *Sirt3* exacerbated increased XBP1s acetylation and transcription of downstream cytokines. **(A)** BMDMs derived from WT and *Sirt3* ^-/-^ mice were stimulated with 20μM IMQ for 0, 3, 6, and 9 hours and total cell lysates were immunoprecipitated with anti-XBP1s antibodies; the overall XBP1s levels and its acetylation levels were examined by western blots. **(B)** mRNA levels of inflammatory cytokines were determined by qRT-PCR. Values are expressed as mean ± SEM. *: P<0.05, **: P<0.01, ***: P<0.001, ****: P<0.0001. IMQ, imiquimod; IP, immunoprecipitation; Ac-K, Acetylated-Lysine. WT, wild type; KO, *Sirt3* knockout.

To exclude the possibility of nonspecific effects of 3-TYP, we next assessed whether the deletion of *Sirt3* in BMDMs aggravated the TLR7/8-XBP1s response. BMDMs derived from *Sirt3*-KO and wild mice were stimulated with IMQ. As expected, *Sirt3* deficiency resulted in elevated total expression and acetylation of XBP1s in *Sirt3*-KO BMDMs compared to the WT group (**Figure 3A**). Moreover, *Sirt3* deletion upregulated the transcription of XBP1s-dependent inflammatory cytokines, including *Il23*a*, Il6* and *Tnf*a** (**Figure 3B**).

### 3.4 The modulation effect of SIRT3 on TLR7/8 response is mediated by XBP1s

**Figure 4:**
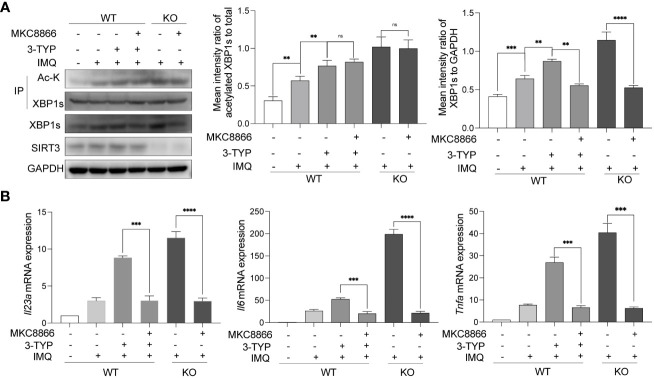
SIRT3 modulated TLR7/8 response through XBP1s. **(A)** WT and *Sirt3^-/-^* BMDMs pretreated with or without 50μM 3-TYP for 1 hour and 20μM MKC8866 for 24 hours were stimulated with 20μM IMQ for 9 hours and total cell lysates were immunoprecipitated with anti-XBP1s antibodies; the overall XBP1s levels and its acetylation levels were examined by western blots. **(B)** mRNA levels of inflammatory cytokines were determined by qRT-PCR. Values are expressed as mean ± SEM. ns: P>=0.05, **: P<0.01, ***: P<0.001, ****: P<0.0001. IMQ, imiquimod; IP, immunoprecipitation; Ac-K, Acetylated-Lysine; WT, wild type; KO, *Sirt3* knockout.

In order to further establish a causal link between SIRT3 and XBP1s in psoriasis, we performed a rescue experiment by pharmacologically decreasing XBP1s level with MKC8866. MKC8866 is an optimized IRE1α RNase-specific inhibitor that inhibits XBP1s expression (15). Compared with the control groups, MKC8866 treated SIRT3-inhibited or *Sirt3*-KO BMDMs showed a significant decrease in XBP1s expression after IMQ application (**Figure 4A**). In addition, MKC8866 downregulated the expression of inflammatory cytokines including *Il23*a*, Il6* and *Tnf*a** in SIRT3-inhibited or *Sirt3*-KO BMDMs stimulated by IMQ (**Figure 4B**). These results suggested that the modulation of TLR7/8 response by SIRT3 was mediated through XBP1s.

### 3.5 SIRT3 regulates the pathogenesis of psoriasis

**Figure 5:**
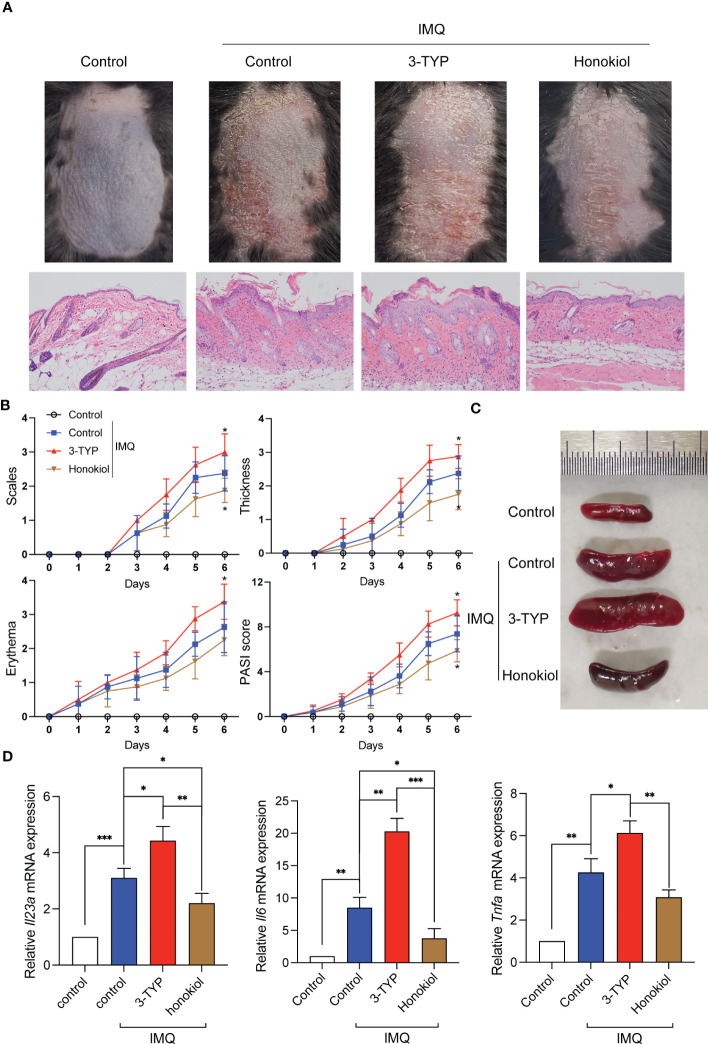
SIRT3 upregulation or downregulation affected IMQ-induced psoriasiform skin inflammation. **(A)** Representative pictures and H&E staining of mice back skins on day 7 from C57BL/6 mice pretreated with 3-TYP or Honokiol or neither were applied a daily topical dose of 62.5 mg of IMQ cream on the shaved back for 7 consecutive days. **(B)** The severity of psoriatic response in back skin, including scaling, erythema, and thickness in IMQ-treated mice, was measured daily. (n =8). **(C)** The representative picture of spleen sizes in each group. **(D)** mRNA levels of inflammatory cytokines in the back skin were determined by qRT-PCR. Values are expressed as mean ± SEM. *: P<0.05, **: P<0.01, ***: P<0.001. IMQ, imiquimod; WT, wild type.

To verify the functional relevance of SIRT3 upregulation or downregulation in the development of psoriasis, we inhibited or activated SIRT3 activity pharmacologically with 3-TYP or Honokiol and then constructed an IMQ-induced psoriasis mouse model. Honokiol has been applied in many studies as a pharmaceutical SIRT3 activator (16). Scaling, erythema, and skin thickening were gradually observed on the back of the mice after 7 days of IMQ treatment. Compared to the control group, the 3-TYP group displayed accelerated clinical manifestations and pathological alterations characteristic of psoriasis-like skin lesions, while the honokiol group exhibited attenuated phenotypic and pathological features of psoriatic skin lesions (**Figures 5A, B**). Systemic inflammatory effects induced by IMQ treatment lead to splenomegaly, another characteristic feature of this mouse model (17). We observed that mice treated with 3-TYP displayed more significant splenomegaly, whereas the honokiol group showed alleviated splenomegaly compared to the control group (**Figure 5C**).

To assess whether SIRT3 mediates the cytokine levels in the lesions, we extracted the cytokine mRNA derived from the lesions and determined the expression levels by qRT-PCR analysis. The results demonstrated that *Il23*a*, Il6* and *Tnf*a** mRNA expression of the 3-TYP group was significantly increased compared to the control group, and the honokiol group showed a reduction in the increase of cytokine mRNA levels upon IMQ stimulation to a certain extent (**Figure 5D**). These results demonstrated that SIRT3 downregulation aggravated psoriatic inflammation induced by IMQ, which was attenuated by SIRT3 upregulation.

### 3.6 Deletion of *Sirt3* exacerbates IMQ-induced psoriasis-like response

**Figure 6:**
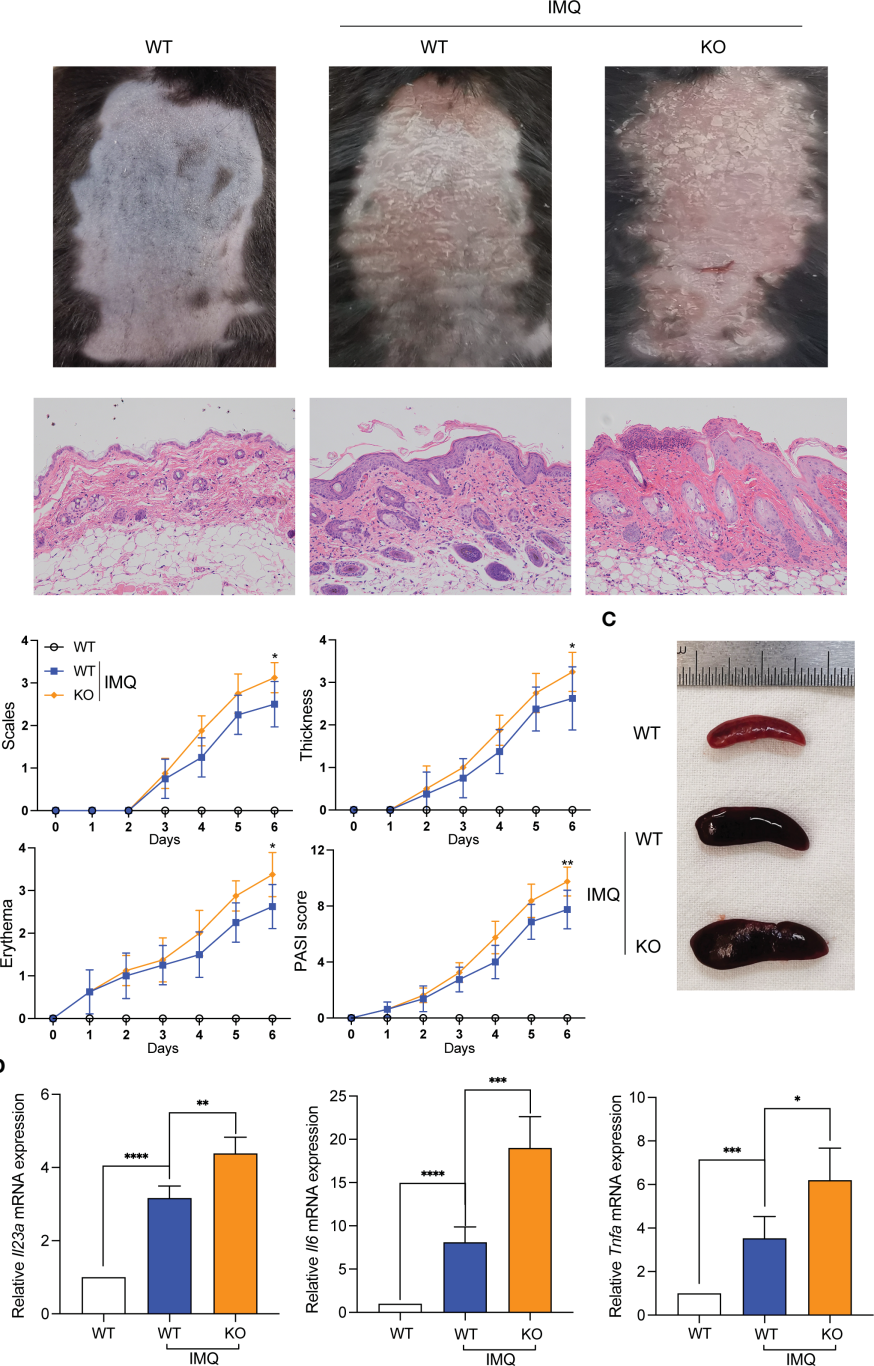
*Sirt3* knockout exacerbated IMQ-induced psoriasiform skin inflammation. **(A)** Representative pictures and H&E staining of mice back skins on day 7. **(B)** The severity of psoriatic response in back skin, including scaling, erythema, and thickness in IMQ-treated mice, was measured daily. (n=8). **(C)** The representative picture of spleen sizes in each group. **(D)** mRNA levels of inflammatory cytokines in the back skin were determined by qRT-PCR. Values are expressed as mean ± SEM. *: P<0.05, **: P<0.01, ***: P<0.001, ****: P<0.0001. IMQ, imiquimod; WT, wild type; KO, *Sirt3* knockout.

We constructed IMQ-induced psoriasis-like mouse models with *Sirt3* ^-/-^ mice to rule out putative unspecific effects of pharmacological inhibitors. The psoriasiform symptoms (scaling, erythema, and skin thickening) in *Sirt3* ^-/-^ mice were similar to those in WT mice but more severe, consistent with our hypothesis (**Figures 6A, B**). The splenomegaly of mice from the *Sirt3* ^-/-^ group was more significant (**Figure 6C**). Besides, The mRNA levels of *Il23*a**, *Il6* and *Tnf-*a** were significantly higher in the *Sirt3^-^* ^/-^ mice lesions (**Figure 6D**).

### 3.7 Decreased SIRT3 in macrophages of psoriatic patients leads to increased XBP1s expression and its acetylation level

**Figure 7:**
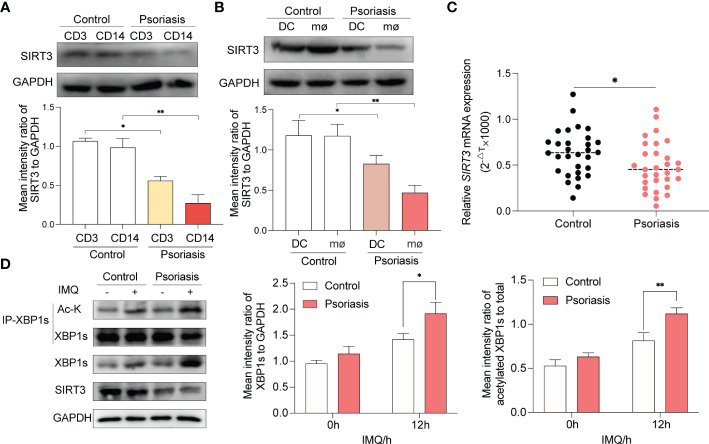
Decreased SIRT3 and increased XBP1s acetylation in macrophages from psoriasis patients. **(A)** CD3+ T cells and CD14+ monocytes from PBMCs of patients with psoriasis and age- and sex-matched healthy volunteers were isolated and SIRT3 levels were examined by western blots. **(B)** PBMCs of patients with psoriasis and age- and sex-matched healthy volunteers were differentiated to DCs and macrophages, and SIRT3 levels were examined by western blots. **(C)** mRNA levels of *SIRT3* in macrophages from patients with psoriasis and age- and sex-matched healthy volunteers were determined by qRT-PCR. **(D)** Macrophages derived from patients with psoriasis and age- and sex-matched healthy volunteers were stimulated by IMQ for 12 hours; XBP1s, and its acetylation level were examined by western blots. Values are expressed as mean ± SEM. *: P<0.05, **: P<0.01. CD3, CD3+ T cells. CD14, CD14+ monocytes. DC, dendritic cell; mø, macrophages; IMQ, imiquimod; Ac-K, Acetylated-Lysine; IP, immunoprecipitation.

In the initial steps of psoriasis pathogenesis, the activation of T cells, macrophages and DCs was thought to be predominant (2). In order to probe the expression of SIRT3 in T cells and monocyes, we isolated CD3+ T cells and CD14+ monocytes from PBMCs of 10 patients with psoriasis and age- and sex-matched healthy volunteers *via* flow cytometry cell sorting technology. We found that SIRT3 was decreased in both T cells and monocytes derived from patients with psoriasis compaired to healthy volunteers, and the difference was more significant in monocytes (**Figure 7A**). Then, monocytes from PBMCs were incubated to be differentiated to DCs and macrophages. Althought both DCs and macrophages from psoratic patients showed a reduced SIRT3 expression, the difference was more significant in macrophages (**Figure 7B**). Subsequently, we found that *SIRT3* mRNA of macrophages from 30 patients with psoriasis decreased compared to paired healthy volunteers (**Figure 7C**). Next, we explored the effect of SIRT3 expression on XBP1s levels and acetylation in macrophages of patients with psoriasis, as shown in **Figure 7D**, after IMQ stimulation, XBP1s showed increased expression and acetylation in macrophages, especially in macrophages derived from psoriatic patients.

## 4 Discussion

In the present study, we found that SIRT3 was essential for modulating psoriasis-like inflammation and was downregulated in psoriatic macrophages. SIRT3 deficiency exacerbated the TLR7/8-XBP1s response *via* deacetylation activity targeting XBP1s, contributing to the transcription of proinflammatory cytokines IL-23, IL-6, and TNF-α and thus promoting psoriasis progression and disease severity.

IL-23 is central to the survival and proliferation of TH17 and TH22 cells and is well-established as a dominant driver in the pathogenesis of psoriasis. Mediated intracellularly *via* Tyk2-Jak2 and STAT3 in TH17, IL-23 signaling leads to the transcription of key inflammatory mediators (2). According to classical theory, IL-23 production is triggered by the NF-κB family, activated when innate immune receptors detect various molecular patterns (18–20). An increasing body of evidence from recently published studies suggests that c-Jun is an essential downstream transcription factor regarding IL-23 in TLR7/8-stimulated DCs in psoriatic skin inflammation (21). Similarly, CHOP and XBP1 in UPR play a nonnegligible role in generating IL-23 (4, 22). Our study found that XBP1s expression was increased upon TLR7/8 activation by IMQ, which correlated with an increase in *Il23*a** mRNA level. This suggested that the upregulation and maturation of XBP1s were significant in amplifying the IL-23-mediated inflammatory response and that targeting XBP1s could be a potential therapeutic intervention for IL-23-dependent inflammatory diseases.

Acetylation and deacetylation are the most studied post-translational modifications of XBP1s (7, 13, 23), constituting an essential mechanism in regulating protein levels of XBP1s and its transcriptional activity. Given that the deacetylase activity of SIRT3 also affected XBP1s acetylation and the downstream cytokines, we focused on the deacetylation activity of SIRT3. Much emphasis has been placed on exploring the deacetylation ability of SIRT3 in regulating inflammation in macrophages in recent years. Previous studies have primarily focused on mitigating the proinflammatory activation of the NLRP3 inflammasome and promoting the activation of the NLRC4 inflammasome to yield protective effects (24–27). Our data showed that the anti-inflammatory effect of SIRT3 was mediated by its deacetylation activity on XBP1s, and SIRT3 regulated the development of psoriasis-like skin inflammation in an IMQ-induced psoriasis-like mouse model, which provided novel insights that SIRT3 regulates inflammation through XBP1s in UPR independent of the inflammasome. Besides, SIRT3 governs the production of IL-23 and TNF-α, dominant cytokines in the pathological processes in psoriasis, endowing SIRT3 with higher status in psoriasis than in other inflammatory diseases.

The present study focused on the effect of SIRT3 in the nucleus, although the intracellular localization of SIRT3 is still a subject of debate. Most studies about SIRT3 reported its deacetylation effect in the mitochondria, and increasing evidence suggests that SIRT3 is exclusively localized in the mitochondria (28, 29). However, there is a growing consensus that the SIRT3 is localized in the nucleus, exhibiting several biological functions (30–35). A recent report revealed that SIRT3 interacted with the nuclear envelope and heterochromatin-associated proteins (34). In this study, we provide hitherto undocumented evidence that SIRT3 interacts with an inflammation-related nuclear transcription factor, XBP1s, which can amplify the inflammatory cascade. More importantly, SIRT3 indirectly regulates IL-23, IL-6, and TNF-α *via* XBP1s, indicating that SIRT3 prevents excessive inflammation activation at the transcription level, which is critical for protection against inflammatory damage.

Although we confirmed that SIRT3 plays a role in TLR7/8-mediated inflammation by regulating XBP1s, its functions in other inflammation pathways cannot be ignored. Nuclear factor-κB (NF-κB), the classical nuclear factor mediating regulation of TLR-mediated inflammation, was considered a direct substrate of SIRT3. Reduced SIRT3 expression is associated with elevated NF-κB phosphorylation and acetylation, which account for the significant downregulation of inflammatory markers (36). Besides, SIRT3 reportedly yields an anti-inflammatory effect by decreasing nuclear NF-κB p65 expression (37). Moreover, SIRT3 activation has been reported to have protective effects against renal tubulointerstitial fibrosis induced by unilateral ureteral obstruction through the NF-κB/transforming growth factor-β1/Smad signaling pathway (13). IL-23, IL-6, and TNF-α are known targets of NF-κB signaling upon TLR activation (38). Nonetheless, whether the anti-inflammatory effect of SIRT3 in macrophages is partially mediated by other signaling pathways, such as TLR-NF-κB, remains unclear, warranting further research.

To our knowledge, this is the first study to reveal that SIRT3 functions in synthesizing IL-23 through the TLR7/8-XBP1s pathway. The present study provides evidence that the acetylation of XBP1s in macrophages aggravates the inflammatory response *via* the transcription of downstream cytokines. SIRT3 plays a critical role in regulating the acetylation status of XBP1s, which governs immune dysregulation and the development of psoriatic pathologies dependent on IL-23. In conclusion, the deacetylation function of SIRT3 targeting XBP1s represents a new regulatory mechanism in inflammation and provides a latent target for treating psoriasis.

## Data availability statement

The original contributions presented in the study are included in the article/**Supplementary Material**. Further inquiries can be directed to the corresponding authors.

## Ethics statement

The studies involving human participants were reviewed and approved by Medical Ethics Committee of Shanghai Sixth People’s Hospital. The patients/participants provided their written informed consent to participate in this study.The animal study was reviewed and approved by Animal Experiment Welfare Ethics Committee of Shanghai Sixth People’s Hospital.

## Author contributions

MG, HZ and WL performed the experiments, analyzed the data, and wrote the paper. HD designed the study. S-MD, YS, QM, NL participated in its design and helped to draft the manuscript. MW gave valuable suggestions about the study. All authors contributed to the article and approved the submitted version.
