# Supplementary material for: SIRT3 alleviates imiquimod-induced psoriatic dermatitis through deacetylation of XBP1s and modulation of TLR7/8 inducing IL-23 production in macrophages
Source: Front Immunol. 2023 May 19;14:1128543. doi: 10.3389/fimmu.2023.1128543 (PMC10235469; doi:10.3389/fimmu.2023.1128543)
Supplement: Supplementary file 1 [file DataSheet_1.pdf]

Supplementary Figure 1

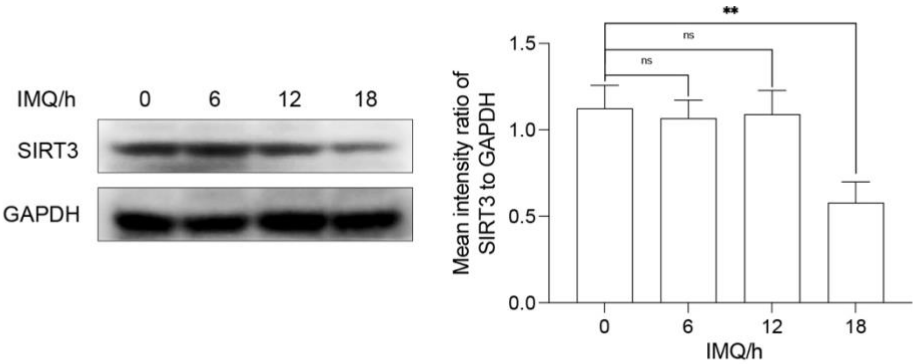

Supplementary Figure 1. The expression of SIRT3 upon IMQ stimulation. BMDMs were stimulated with 20μM IMQ for 0, 6, 12, and 18 hours and cell lysates were immunoblotted for SIRT3 and GAPDH. Values are expressed as mean±SEM. ns: P＞=0.05, **: P<0.01. IMQ, imiquimod.
